# Spinal Cord Compression As the Initial Manifestation of Relapsed Acute Myeloid Leukemia: A Case Report and Literature Review of a Rare Presentation

**DOI:** 10.7759/cureus.81509

**Published:** 2025-03-31

**Authors:** Raul Gregg-Garcia, Hamid Sayar

**Affiliations:** 1 Medicine, Indiana University School of Medicine, Indianapolis, USA; 2 Hematology and Oncology, Indiana University School of Medicine, Indianapolis, USA

**Keywords:** acute myeloid leukemia, cns neoplasm, extramedullary disease, myeloid sarcoma, spinal cord compression

## Abstract

Myeloid sarcoma (MS) is a solid mass of myeloid blasts outside the bone marrow (BM). Most cases occur in the setting of intramedullary acute myeloid leukemia (AML), but it can also present in the absence of overt BM disease, as a presentation of newly diagnosed or relapsed AML, or as a progression of myeloproliferative neoplasms or myelodysplastic syndromes. There are a few reports of spinal cord compression due to MS, and there is no consensus regarding its management. Here, we present a case of relapsed AML in the form of MS resulting in spinal cord compression and provide a comprehensive literature review of previously reported cases of MS causing cord compression.

An 18-year-old male was diagnosed with AML with poor-risk cytogenetics in September 2023. He received induction chemotherapy (CTX) with 7+3, followed by consolidation with high-dose cytarabine, achieving remission. He was referred for BM transplant evaluation but opted against it. One year later, he presented with a four-month history of bilateral motor and sensory deficits along with bladder dysfunction. A magnetic resonance imaging (MRI) of the spine showed multilevel nerve root thickening and enhancement and multiple extramedullary masses. Spinal radiation therapy and corticosteroids were given; a biopsy was deferred due to high procedural risks. Given his prior history of AML, the findings were highly suspicious for MS. A complete blood count (CBC) and smear did not show circulating blasts, and a BM exam was inconclusive. Induction CTX with MEC regimen (mitoxantrone, etoposide, cytarabine) was started. A lumbar puncture with CSF flow cytometry confirmed central nervous system involvement with myeloid blasts, and a brain MRI revealed leptomeningeal disease. Intrathecal CTX was given. A spine MRI on day 15 post-induction showed partial improvement in spinal disease. The patient was discharged 30 days after receiving induction CTX, and he planned to continue his care at a local cancer institute in his home state.

## Introduction

Myeloid sarcoma (MS), previously called granulocytic sarcoma or chloroma, is the name of an extramedullary accumulation of malignant blasts of myeloid lineage that form a solid tumor in non-hematopoietic tissues, altering their normal architecture. In alignment with this definition, MS does not include leukemic infiltration of the liver, spleen, lymph nodes, pleural and pericardial fluid, cerebrospinal fluid (CSF), and non-solid mass tissue infiltration commonly seen in skin, gums, retina, and lungs. Most cases of MS present in the setting of underlying intramedullary acute myeloid leukemia (AML); however, it can also present de novo in the absence of intramedullary disease, as a manifestation of newly diagnosed or relapsed AML, or as progression of myelodysplastic syndromes (MDS) or myeloproliferative neoplasms (MPN) [1-3]. As of now, the Fifth Edition of the WHO classification of hematolymphoid tumors and the International Consensus Classification (ICC) of Myeloid Neoplasms and Acute Leukemias proposes MS as a unique clinical presentation within the category of AML, and although listed separately, all cases of MS should be approached similar to AML and investigated to classify them into a more specific AML subtype [4,5].

Circulating malignant myeloid blasts can virtually disseminate to any extramedullary site, and there have been reports of MS in many different anatomic locations. The most frequently reported anatomic sites affected by MS are connective and soft tissues, breast, and the digestive system; these locations account for approximately 50% of all MS cases [1]. The clinical presentations of MS are diverse and mainly depend on its anatomic location, where it can cause mass effect-related manifestations or even lead to organ dysfunction [6]. Spinal intra- and extramedullary disease is an infrequent presentation of MS, with less than 100 reported cases in the literature. These cases usually present with signs and symptoms of spinal cord compression, representing a medical emergency as it can lead to permanent neurologic deficits, including paralysis/paresis, sensory losses, and bladder and bowel dysfunction. There has not been consensus regarding adequate management of spinal cord compression caused by MS, given its rarity and lack of prospective randomized control trials (RCTs), and the scarce available evidence comes from relatively few published case reports. Most of the latter favor a combination of systemic chemotherapy (CTX), radiation therapy (RT), corticosteroids (CS), and decompressive surgery. Hereby, we present the case of a young adult who presented with signs and symptoms of spinal cord compression approximately one year after completing induction CTX for AML, along with a comprehensive literature review of available data regarding previously reported cases of spinal cord compression caused by MS.

## Case presentation

An 18-year-old male presented to a community hospital in September 2023 with left-sided neck and mandible swelling associated with night sweats. Initial workup was remarkable for a complete blood count of peripheral blood with a WBC of 37.0 K/mm^3^ with 11% of monocytes and 41% of undifferentiated blasts; a CT scan reported bilateral submandibular, anterior cervical, axillary, and inguinal lymphadenopathies. A cervical lymph node core biopsy reported the presence of atypical proliferation of medium-to-large cells with round-to-irregular nuclei and fine chromatin, with CD13+, CD33+, and lysozyme+ on flow cytometry and immunohistochemistry, consistent with lymph node involvement by myeloid blasts. He was subsequently referred to our institution. At presentation, he had significant leukocytosis of 40.0 K/mm^3^, and flow cytometry of peripheral blood reported a prominent (55%) blast population characterized by the expression of CD34, CD117 (partial), CD13, CD33, CD123, HLA-DR, CD38, TdT, CD7, and minimal expression of myeloperoxidase (MPO), with negativity for B-cell and other T-cell markers, immunophenotypically consistent with AML. Chromosome analysis reported a complex profile 46,XY,del(1)(p36.1),del(9) (q13q22),-10,inv(11)(q21q23),-12,-16,+mar1-3 in 18 out of 20 analyzed cells. Fluorescence in situ hybridization (FISH) analysis on the peripheral blood reported loss of 16q22.1 (CBFB), loss of 12p13.2 (ETV6), and gain of 19p13.3 (TCF3). Next-generation sequencing (NGS) reported MLLT10, PHF6, and PTPNH mutations. Given the high percentage of myeloid blasts in the peripheral blood, a bone marrow (BM) examination was deferred, and the patient received induction CTX with 7+3 regimen (seven days of cytarabine and three days of idarubicin). A BM examination on day 15 of induction revealed a hypocellular BM (<10%) with mildly increased blasts (approximately 10%). Given these findings and his poor-risk cytogenetics, the patient was recommended to receive high-dose cytarabine (HiDAC) for re-induction; however, he declined and opted to be discharged once he would not be transfusion-dependent. A subsequent BM examination performed as outpatient on day 30 after induction CTX reported maturating trilineage hematopoiesis with left-shifted myelopoiesis and no increase in blasts, consistent with complete morphologic remission; flow cytometry also reported a heterogeneous cell population and no increase in blasts; cytogenetics reported a karyotype (46, XY). The patient received one cycle of consolidation CTX with HiDAC and was referred for stem cell transplantation (SCT) consideration. However, the patient decided not to pursue a transplant due to concerns about potential side effects. Subsequently, he did not return to the clinic and was lost to follow-up.

**Figure 1:**
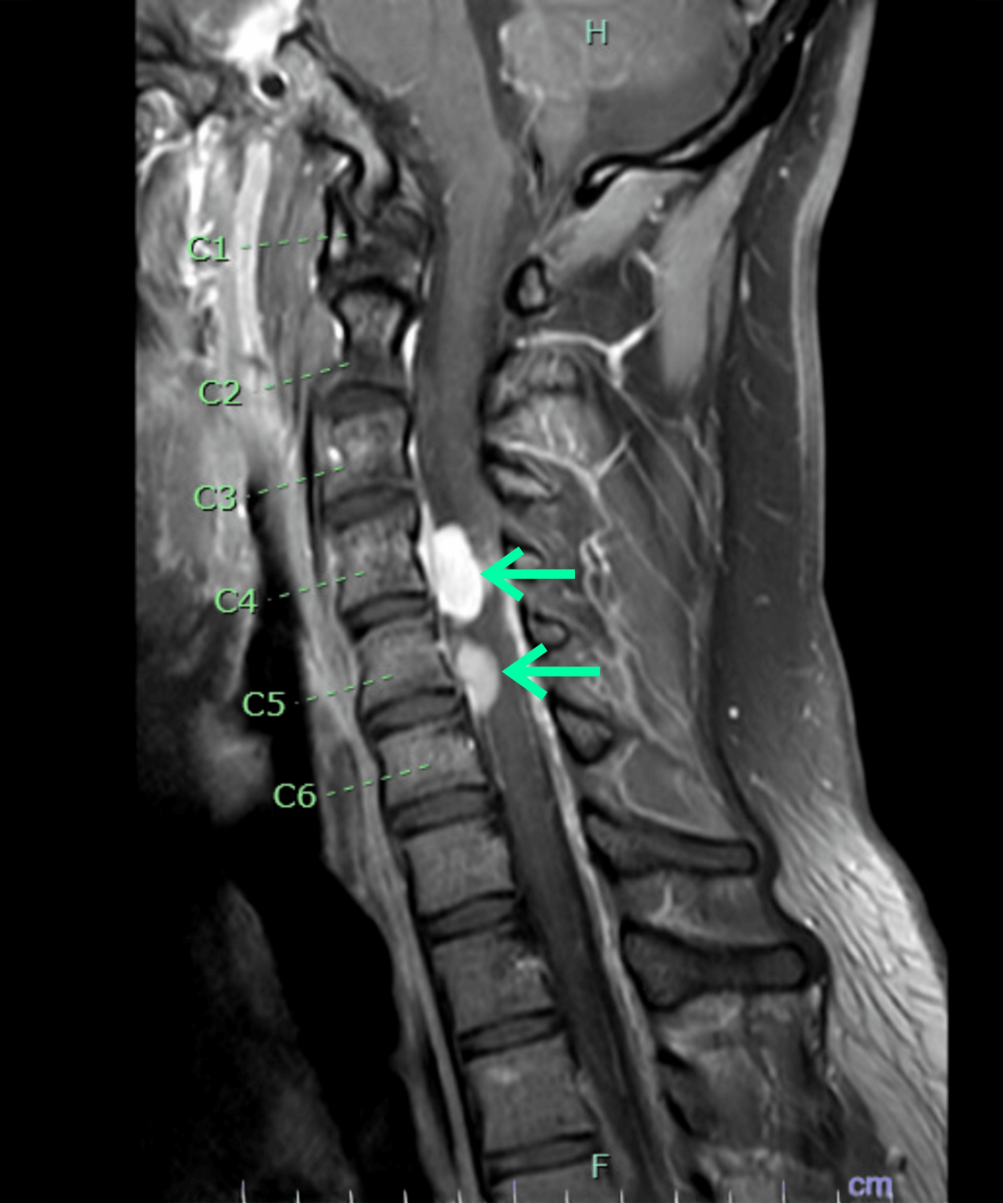
Cervical spine magnetic resonance imaging (MRI) showing two areas of tumor infiltration (myeloid sarcoma) involving the intradural extramedullary spaces (green arrows), measuring 18 mm and 13 mm at their largest dimensions.

In September 2024, the patient presented to a community hospital with a four-month history of slowly progressive right upper extremity paresthesia associated with posterior neck and mid-low back pain that eventually advanced to severe sensory deficits and paresis of both lower extremities and limited ambulation, along with urinary retention and constipation. He also had a 35-lb unintentional weight loss over three to four months. A whole spine MRI reported multilevel nerve root thickening and enhancement from C1 to T3 and L5 to S3, as well as multiple enhancing intradural extramedullary masses (Figure 1). The cervical lesions were causing significant thecal sac stenosis and cord compression, and the cauda equina was thickened with multiple foci of enhancement. The patient received three days of RT along with high-dose CS. Neurosurgery and neuro-interventional radiology were consulted to consider a potential biopsy of spinal masses; however, this was deferred as they considered it a high-risk procedure with a very high likelihood these lesions were representing MS in the setting of previously treated AML. He was then referred again to our institution.

**Figure 2:**
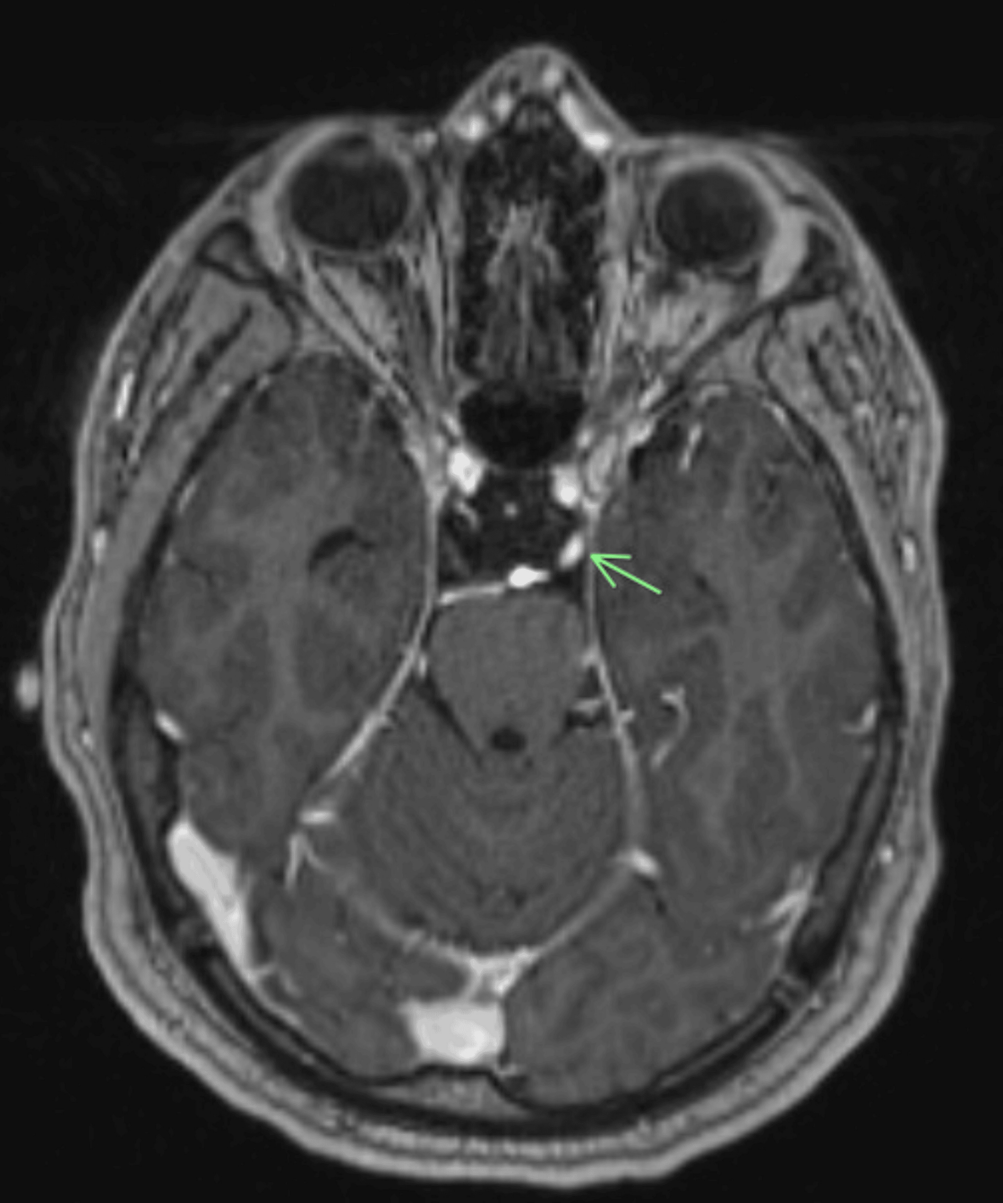
Brain magnetic resonance imaging (MRI) showing abnormal enhancement along the left oculomotor nerve (green arrow), raising concern for leptomeningeal disease.

Following RT, the patient reported partial improvement in his neurologic deficits. Physical examination revealed paresis and hypoesthesia in his right arm and bilateral lower extremities, with the right worse than the left. A complete blood count (Table 1) reported hemoglobin (Hgb) 12.0 g/dL, WBC 3.9 K/mm^3^, and platelet count 99 k/mm^3^, with a normal differential count and no circulating blasts. A computed tomography (CT) scan with intravenous contrast of the chest, abdomen, and pelvis showed no disease in these locations. A BM aspiration was performed, and he was started on systemic CTX for induction with MEC regimen (mitoxantrone, etoposide, and cytarabine) for five days. Unfortunately, the BM aspirate sample was inadequate for evaluation. His strength and sensation improved progressively over the next several days, and he could walk with a cane. On day 5 post-induction, a lumbar puncture for CSF analysis and intrathecal (IT) CTX (methotrexate, cytarabine, hydrocortisone) administration was performed. The CSF contained myeloid blasts, confirmed by cytology and flow cytometry, and was consistent with central nervous system (CNS) involvement by AML. A subsequent brain MRI showed abnormal enhancement along the left oculomotor nerve (Figure 2), raising concern for leptomeningeal disease. A repeat whole-spine MRI on day 15 post-induction reported a partial response demonstrated by shrinking the intraspinal masses, spine nerve roots, and cauda equina thickening. After improving the platelet count, he received another dose of IT CTX. After the patient achieved count recovery, he was discharged with the plan to continue his care close to home.

**Table 1:** Laboratory findings. BUN: blood ureic nitrogen; CBC: complete blood count; CTX: chemotherapy; RBC: red blood cells; WBC: white blood cells

Test	Sep 2023 (at initial presentation)	Oct 2023 (on discharge after induction CTX)	Sep 2024 (at presentation)	Oct 2024 (after re-induction CTX)	Reference values
CBC with differential					
WBC	37.0 x10^3^/µL	1.5 x10^3^/µL	3.9 x10^3^/µL	1.4 x10^3^/µL	3.6 x10^3^-10.6 x10^3^/µL
Neutrophils (%)	4	8	62	69	40-60
Band form (%)	2	0	5	4	0-3
Lymphocytes (%)	41	81	24	19	20-40
Monocytes (%)	11	10	6	5	2-8
Blasts (%)	41	0	0	0	0
RBC	4.22 x10^6^/µL	2.49 x10^6^/µL	3.9 x10^6^/µL	3.0 x10^6^/µL	4.18 x10^6^-5.51 x10^6^/µL
Hemoglobin	13.1 g/dL	7.5 g/dL	12 g/dL	9.1 g/dL	13.7-17.0 g/dL
Hematocrit (%)	39.1	21.3	34.5	26.4	40-54
Platelet count	279 x10^3^/µL	108 x10^3^/µL	99 x10^3^/µL	95 x10^3^/µL	150 x10^3^-450 x10^3^/µL
Basic metabolic panel					
Sodium	139 mEq/L	138 mEq/L	135 mEq/L	136 mEq/L	135-145 mEq/L
Potassium	4.4 mEq/L	3.3 mEq/L	4.0 mEq/L	3.5 mEq/L	3.5-5.5 mEq/L
Chloride	101 mEq/L	103 mEq/L	101 mEq/L	101 mEq/L	98-108 mEq/L
Bicarbonate	32 mEq/L	25 mEq/L	26 mEq/L	26 mEq/L	22-29 mEq/L
Creatinine	0.6 mg/dL	0.65 mg/dL	0.67 mg/dL	0.57 mg/dL	0.8-1.4 mg/dL
BUN	15 mg/dL	7 mg/dL	20 mg/dL	3 mg/dL	0.5-20 mg/dL

## Discussion

Materials and methods

We conducted a comprehensive review of cases of MS presenting with signs and symptoms of spinal cord compression reported in the literature. We performed a literature review in November 2024 using PubMed as a database. We included case reports and case series articles that included one of the following search terms in their titles or abstracts: myeloid sarcoma, acute myeloid leukemia, granulocytic sarcoma, myeloblastoma, or chloroma, in combination with either one of the following search terms: spinal cord compression, myelopathy, paraplegia, or hemiplegia.

We found 155 articles that met our initial search criteria. We excluded non-English articles and those unavailable as full-text online articles. We also excluded articles that were not case reports or case series of MS presenting with manifestations of spinal cord compression.

Results

A total of 62 case reports and case series articles (making a total of 69 cases) were included in this literature review. A summary of these reported cases from 1973 to 2023 is presented in Table 2. Demographic and patients' characteristics are presented in Table 3. Fifty-five (80%) of the reported cases were male, and the median age at presentation was 29 years (14-47 years). Only 22 (32%) of patients had a known prior history of hematologic diseases such as AML (the most common one reported), MPN, or MDS. The most frequently reported clinical manifestations were paresis/paralysis (81%), sensory deficits (59%), back pain (59%), bladder dysfunction (40%), and bowel dysfunction (26%). Fifty-seven percent of cases were found to have BM disease at the time of presentation. Still, only 11% of cases reported CNS involvement (demonstrated by the presence of myeloid blasts on CSF analysis). Regarding management, patients mostly underwent systemic CTX (71%), RT (56%), and/or surgery (56%); other reported treatment strategies included IT CTX (19%) and SCT (13%). The median follow-up period between the initial presentation and the last evaluation before writing the case report was six months (3-12 months), and 46% of the patients were alive at the time of the report.

**Table 2:** Literature review of patients with spinal cord compression due to MS sarcoma. AML: acute myeloid leukemia; ARDS: acute respiratory distress syndrome; ATRA: all-trans retinoic acid; BM: bone marrow; BMT: bone marrow transplant; CML: chronic myeloid leukemia; CNS: central nervous system; CS: corticosteroids; CTX: chemotherapy; DIC: disseminated intravascular coagulation; DLI: donor lymphocyte infusion; HiDAC: high-dose cytarabine; IT: intrathecal; L: left; LE: lower extremity; LP: lumbar puncture; MDS: myelodysplastic syndrome; MPN: myeloproliferative neoplasm; NHL: non-Hodgkin lymphoma; PMF: primary myelofibrosis; R: right; RT: radiotherapy; SCT: stem-cell transplant; TKI: tyrosine kinase inhibitor; UE: upper extremity

First author, year, reference	Sex, age	History of AML or MPN	Clinical presentation	Anatomic location	CNS disease at presentation	BM disease at presentation	Flow cytometry, cytogenetic and genetic hallmarks	Management	Interval follow-up	Outcome at time of publication
Stefansson, 1973 [7]	F, 11	No	Acute lumbar pain, paraplegia, sphincter disturbances	T7 epidural mass	No	Yes	Not mentioned	Surgery, IT methotrexate, RT, and systemic CTX (purinethol, rubidomycin, cytarabine, prednisone)	Not mentioned	Alive. Partial response to treatment with minor improvement in the paraplegia
Hildebrand, 1980 [8]	M, 25	Yes, AML	Rapidly progressive paraplegia and complete loss of sphincter control	T3-7 epidural mass	No	No	Not mentioned	Surgery, RT	1 month	Died from pulmonary embolism
Petursson, 1981 [9]	M, 21	No	Flu-like symptoms, hepatosplenomegaly, constipation, urinary retention, perineal sensory deficits, and sacral pain	L5-S1 mass	No	Yes	Not mentioned	RT, and systemic CTX (induction with daunorubicin and cytarabine, followed by re-induction and consolidation with vincristine, prednisone, cytarabine, and 6-thioguanine). Patient relapsed and underwent BMT	4 months	Died from interstitial pneumonia following BMT
Kellie, 1984 [10]	M, 8	No	Low back pain, bilateral LE dysesthesias and paresis	T4-6 epidural mass	No	Yes	Karyotype 45X,-Y, t(8;21)(q22;q22)	Surgery, systemic CTX (induction with cytarabine, 6-thioguanine, and daunorubicin, followed by consolidation with cyclophosphamide, doxorubicin), and IT CTX (cytarabine)	5 months	Alive. Complete remission and resolution of neurologic deficits
Abe, 1986 [11]	M, 17	No	Lumbar pain, paresthesias below the umbilicus, spastic paraplegia	T5-8 epidural mass	No	Yes	t(8;21)	Systemic CTX (two cycles of daunorubicin, cytarabine, 6-mercaptopurine, and prednisolone). Due to first relapse, patient had a course of systemic daunorubicin and enocitabine, followed by surgery (laminectomy with tumor resection) and one cycle of aclarubicin and enocitabine	1 year and 6 months	Died after second relapse
Wodzinski, 1988 [12]	M, 14	No	Paraplegia, T5 sensory level. Second relapse was associated with multiple 2-cm fleshy skin lesions that were demonstrated to be MS	T3-5 epidural mass	No	Yes	Karyotype 45X,-Y, t(8;21)(q22;q22)	Systemic CTX (daunorubicin, cytarabine, and 6-thioguanine), and surgery (laminectomy). Due to first relapse, patient received re-induction with mitoxantrone, cytarabine, and 6-thioguanine	6 weeks	Died after second relapse
	M, 19	No	Back pain, absent right ankle reflex, and urinary retention	T7-11 paraspinal mass	No	Yes	Karyotype 45X,-Y, t(8;21)(q22;q22)	IT methotrexate, systemic CTX (daunorubicin, cytarabine, and thioguanine), and spine RT. Due to first relapse, patient received re-induction with mitoxantrone and cytarabine, followed by BMT	6 months	Died after BMT
	M, 17	Yes, AML	Bilateral LE weakness and urinary retention	T9-10 epidural mass	Not mentioned	Yes	Complex karyotype	Surgery, RT, and systemic CTX	Not mentioned	Not mentioned
Brown, 1989 [13]	M, 12	Yes, AML	Back pain, pleural effusion, post-LP urinary retention, bilateral LE paresis, and T5 sensory level	Paraspinal mid- and lower thoracic spinal mass with extension to the epidural space	Not mentioned	Yes	Not mentioned	CS, thoracic spine RT, and systemic CTX	3 months	Died from persistent BM disease
Ripp, 1989 [14]	M, 58	No	Back pain, paraparesis, T6 sensory level, perianal numbness, bladder and bowel dysfunction	T5-7 and L5-S1 epidural masses	Not mentioned	Yes	Not mentioned	Systemic CTX for presumed multiple myeloma, followed by local RT and induction CTX (daunorubicin, cytarabine, and 6-thioguanine)	3 months	Died from sepsis
Kook, 1992 [15]	M, 10	No	Gradual bilateral LE paresis hyperreflexia, T5 sensory level, loss of sphincter tone.	Epidural thoracic spinal mass	Not mentioned	Yes	MPO+, CD13+, CD33+	Surgery, followed by RT, IT methotrexate, and systemic CTX (vincristine, doxorubicin, cyclophosphamide, CS) for presumed NHL. After BM showed AML, patient received CTX with aclacinomycin, cytarabine, and CS	1 year and 4 months	Died from intracranial hemorrhage
Frohna, 1993 [16]	M, 77	Yes, PMF	Low back pain and unsteady gait that progressed to paraplegia	T5-L2 epidural mass	Not mentioned	Yes	Not mentioned	None	2 weeks	Died before diagnosis
Aizawa, 1996 [17]	M, 19	No	Low back pain, paraparesis, urinary retention, T9 sensory level	T5-7 epidural space	Not mentioned	Yes	MPO+, Karyotype 45X,-Y, t(8;21) (q22;q22)	Surgery, systemic CTX	6 months	Alive. Complete remission and resolution of neurologic deficits
Deme, 1997 [18]	M, 47	No	Neck and arm pain, followed by quadriparesis and ataxia	C6-7 epidural space	Yes	No	CD45+, CD43+, MPO+, lysozyme+, CD20-, CD45RO-	Surgery, RT, IT methotrexate. After first relapse, the patient had systemic CTX with cytarabine followed by allo-SCT	1 year	Died from aspiration related to methotrexate encephalopathy.
	M, 49	No	Low back pain, urinary retention, and right leg paresis and numbness	L3-L4 epidural, paraspinal, and foraminal regions	Not mentioned	Not mentioned	CD45+, CD43+, MPO+, CD20-, CD45RO-	Surgery, RT, and systemic CTX	Not mentioned	Alive. Patient getting CTX. Improvement of neurologic deficits
	M, 15	No	Bilateral LE pain and paresis, 15-lb weight loss	L2 vertebral body with epidural extension	No	No	CD45+, CD43+, MPO+, lysozyme+, CD20-, CD45RO-	Surgery, systemic CTX, followed by autologous SCT. Re-induction CTX was given after relapse but was unsuccessful	1 year	Died after relapse
Sajjad, 1997 [19]	M, 22	No	Urinary retention, loss of anal sphincter tone, bilateral LE sensory deficits, and weight loss	L4 and S1 peridural spaces	Not mentioned	No	Not mentioned	Not mentioned	Not mentioned	Not mentioned
Eser, 2000 [20]	F, 17	No	Left facial nerve palsy, paraplegia, loss of rectal sphincter tone, and T4 sensory level	T4 extramedullary-intradural space	Not mentioned	Yes	LCA+, CD68+, lysozyme+	Surgery and systemic CTX (doxorubicin and cytarabine)	Not mentioned	Died from sepsis after second course of chemotherapy
Ugras, 2001 [21]	M, 13	No	Low back pain, bilateral LE pain, paraparesis, and L1 sensory level	T11-L1 epidural space	Not mentioned	No	MPO+, CD45+, CD68+, CD43, CD3-	Surgery	1 year	Died
Landis, 2003 [22]	M, 29	No	Lower thoracic back pain and soft tissue swelling	T7-9 spine	No	Yes	CD45+, CD43+, CD34+, MPO+, CD117+, CD20-, CD3-, t(8;21)(q22;q22)	Systemic CS, surgery, IT cytarabine and systemic CXT (induction with idarubicin and cytarabine, followed by consolidation with cytarabine)	6 months	Alive. In remission
Hamadani, 2005 [23]	M, 33	No	Thoracic spine pain, left leg paresis, sensory deficits, B-symptoms	T6-8 spine	No	Yes	MPO+, S100+, CD68+, CD3-, CD20-, CD1a-, CD30-	Systemic CXT (induction with daunorubicin and cytarabine, reinduction with HiDAC, and reinduction again with mitoxantrone plus HiDAC), followed by BMT x2 (due to engraftment failure of first BMT)	3 months	Alive. In remission
Kalayci, 2005 [24]	M, 24	No	Low back pain, paraparesis, T5 sensory level, and urinary retention	T3-5 epidural space	Not mentioned	No	MPO+, LCA+, lysozyme+	Systemic CS, surgery, systemic CTX, spine RT	8 months	Alive. In remission
Meltzer, 2005 [25]	M, 10	No	Low back pain, paraparesis, bilateral LE sensory deficits	T2-6 prevertebral area with extension to epidural space, bone cranial involvement	No	Yes	Not mentioned	Systemic CS and CTX, surgery, spine RT	1 year and 6 months	Alive. In remission
Seo, 2006 [26]	M, 59	Yes, AML	Back pain, paraplegia, bilateral LE paresthesias	T8-11 epidural and paravertebral spaces	Not mentioned	No	CD45+, CD43+, CD34+, MPO+, lysozyme+, CD3-, CD20-	CS only	Not mentioned	Died from pneumonia
Widhalm, 2006 [27]	F, 35	No	Paraparesis, T5 sensory level, sphincter dysfunction, and urinary retention	T3-5, and S1-S4 intradural space, right parietal extra- and intracranial regions with invasion of the sensory cortex	Not mentioned	No	CD34+, CD43+, CD117+, MPO+, t(8;21)(q22;q22)	Surgery, systemic CTX (cyclophosphamide) followed by whole-body RT and allo-BMT	7 years	Alive. In remission
Balleari, 2007 [28]	F, 76	No	Large inguino-crural mass, bilateral UE paresis and hypoesthesia	C7-T2 subdural space with extension to the subarachnoid space	Yes	Yes	MPO+, CD33+, CD34+, CD20-	Systemic CTX (induction with fludarabine, cytarabine, and idarubicin)	3 months	Died from pneumonia and ARDS
Chauhan, 2007 [29]	F, 53	Yes, CML	Paraparesis, right T10 sensory level,	T10-12 epidural space	Not mentioned	Not mentioned	Not mentioned	Not mentioned	Not mentioned	Not mentioned
Tsai, 2007 [30]	M, 29	Yes, AML	Back pain, paraparesis, perineal anesthesia, fecal and urinary incontinence	Multiple lumbar subarachnoid lesions + S1-5 subdural space	Not mentioned	Not mentioned	Not mentioned	Regional RT, systemic CS, IT CTX (cytarabine) and systemic salvage CTX	1 month	Died from febrile neutropenia and septic shock
Al-Sobhi, 2008 [31]	F, 23	Yes, AML	Paraplegia	T4-5 and T8-9 paraspinal region	Not mentioned	Yes	t(8;21)	Systemic CS, CTX (re-induction with anthracycline and cytarabine, followed by three cycles of consolidation with HiDAC), and adjuvant RT	1 year and 1 month	Died after second relapse
Amalraj, 2009 [32]	F, 14	No	Back pain, paraparesis, T4 sensory level, urinary symptoms	T3-11 epidural space	No	Yes	Not mentioned	Systemic CS, CTX, and local RT	Not mentioned	Alive. Improvement of neurologic deficits
Olcay, 2009 [33]	M, 12	No	Left cheek swelling, bilateral LE pain, S1 sensory level, fecal and urinary incontinence	Left maxillary sinus, cauda equina nerve roots	No	Yes	CD45+, CD15+, CD33+, CD117+, CD34+, CD13+, CD19+, CD20+	Systemic CTX (induction with cytarabine, mitoxantrone, etoposide, and CS, followed by consolidation with cytarabine and daunorubicin), IT CTX (methotrexate, cytarabine, prednisolone), and regional RT	Not mentioned	Died from pneumonia and DIC
Takeda, 2009 [34]	M, 13	No	Exophthalmos, back pain, paraparesis	Left temporal lobe, left orbit, T5-6 and epidural L5-S5 space	Not mentioned	Yes	MPO+, CD13+, CD33+, CD34+, CD45+, CD56+	Systemic CS, surgery, systemic CXT, and BMT	18 months	Alive. In remission and with complete recovery of neurologic deficits
Verra, 2009 [35]	M, 45	Yes, AML	Back and R leg pain, gait disturbance, R dorsi-flexion weakness. After second relapse, patient had R flank pain with dysesthesias in dermatomes T11-12, and vision loss	L5-S2 intraspinal extra-medullary. Multifocal nerve roots of thoracic spine, and multifocal intracranial lesions	Yes	No	Not mentioned	Surgery, RT, systemic CTX (HiDAC), and DLI. IT CTX (cytarabine) after relapse, followed by second DLI.	3 months	Alive. With refractory disease and persistent neurologic symptoms
Amritanand, 2010 [36]	M, 15	No	Back pain, paraparesis	Epidural T8-10 space	Not mentioned	No	MPO+, CD43+, CD99+, CD20-, CD30-, CD3-	Surgery, RT	9 months	Died
Pacilli, 2010 [37]	M, 38	Yes, AML	Chest pain, paraparesis	Extramedullary intraspinal T6-8	Not mentioned	No	MPO+, CD117+, CD45+, CD34-, CD79a-, CD20-, CD3-, CD15-, CD56-, t(15;17) (q22;q21-q22), PML-RAR𝝰 rearrangement	Surgery, RT, systemic CXT (ATRA and arsenic trioxide)	1 year and 8 months	Alive. In remission
Serefhanoglu, 2010 [38]	M, 22	No	Back pain, paraparesis, eosinophilia	Supratentorial and epidural space of all skull bones, C2-3, C7-T10, and L5 epidural and, prevertebral and paravertebral spaces with involvement of the spinal cord	Yes	No	MPO+, CD34+, CD2-, CD3-, CD5-, CD20-, CD30-, CD56-, Tdt-	Cranial and cervical RT followed by systemic CTX (idarubicin + cytarabine)	Not mentioned	Died from septic shock
	F, 43	No	Left arm and back pain	C6-7 epidural space	Yes	No	MPO+, CD3-, CD5-, CD7-, CD10-, CD20-, Tdt-, normal cytogenetics	Spinal and cranial RT, systemic CTX (mitoxantrone + cytarabine), IT CTX	Not mentioned	Died due to ventricular fibrillation and cardiac arrest
Chargari, 2011 [39]	M, 41	No	Back pain, paraplegia, T10 sensory level, loss of sphincter tone	T8 epidural mass	Not mentioned	No	MPO+, CD20-, CD79a-, CD1a-, CD3-, CD5-	Surgery, spinal RT	1 year	Relapsed with AML and was referred for induction CTX
Baikaidi, 2012 [40]	F, 75	No	Back and L leg pain, gait imbalance	T12 paravertebral mass	Not mentioned	Yes	CD43+, CD117+, CD30+, CD34-, CD20-, CD79a-, TdT-, CD3-, CD2-, hyperdiploid, t(9;22), BCR-ABL rearrangement	Systemic CS, RT, systemic CTX (decitabine), and TKI (imatinib)	Not mentioned	Alive
Cornfield, 2012 [41]	M, 79	Yes, MDS	Back pain, paraparesis, weight loss	T7 epidural and paravertebral masses	Not mentioned	Yes	CD71, glycophorin A, CD117, and vimentin positivity (consistent with erythroblastic sarcoma)	RT and supportive therapy	2 weeks	Died
Kyaw, 2012 [42]	M, 26	No	Back pain, paraplegia, T4 sensory level	T2-4 and T12-L2 epidural masses	No	Yes	MPO+, CD45+, CD13+, CD33+, CD117+, CD64+, CD34-, CD14-, CD19-, CD79a-, CD3-, t(15;17), PML/RAR𝝰 rearrangement	Regional RT, systemic CTX (induction with ATRA and idarubicin followed by consolidation), IT CTX	Not mentioned	Alive. In remission, undergoing maintenance CTX
Chamberlain, 2013 [43]	M, 23	Yes, AML	Left buttock and thigh pain	S2 nerve root mass	Not mentioned	Yes	Not mentioned	Systemic reinduction CTX and whole-body RT followed by allo-BMT	1 year	Alive. In remission, with resolution of neurologic symptoms
Ganapule, 2014 [44]	M, 43	No	Back pain, paraplegia with bladder and anal sphincter dysfunction	T6-12 paravertebral lesions	Not mentioned	Yes (CML on chronic phase)	MPO+, CD43+, CD34+, CD20-, CD3-, t(9;22), BCR-ABL rearrangement	Hydroxyurea, spinal RT, and TKI (imatinib)	2 months	Alive. With complete hematologic response
Isshiki, 2014 [45]	M, 59	No	Chest pain, bilateral LE pain and paresthesias, paraplegia, bowel and bladder incontinence	T7-10 epidural mass	Yes	Yes	CD25+, CD33+, CD34+, CD3-, CD13-, CD19-, CD20-, FLT3-ITD mutation, karyotype 46,XY, dup(1)(q21;q23)	IT CTX (methotrexate, cytarabine, CS), systemic CTX (induction and consolidation with HiDAC)	4 months	Alive
Kurian, 2014 [46]	M, 39	No	Paraplegia, bladder involvement, T6 sensory level	T6 epidural mass	Not mentioned	No	MPO+, CD117+, CD68+, CD34-, CD56-	Surgery, systemic CTX (daunorubicin + cytarabine)	Not mentioned	Died from sepsis
Joseph, 2015 [47]	M, 20	Yes, AML in the setting of Shwachman-Diamond syndrome	Back pain, paraparesis, T7 sensory level, urinary retention	T4-9 epidural mass	Not mentioned	Not mentioned	Weak CD68+, partial CD43 and CD117	Surgery, RT	5 months	Died from liver failure due to suspected leukemic infiltration
Krishnan, 2015 [48]	M, 22	No	Back pain, gait ataxia, paraparesis, T10 sensory deficit	T6-8 epidural mass	Not mentioned	No	MPO+, CD43+, CD3-, S100-	Systemic CTX for initially suspected lymphoma followed by CS, surgery, and RT due to worsening neurologic deficits. Systemic CTX with fludarabine and cytarabine after diagnosis of MS	2 years	Alive. In remission with resolution of all neurologic deficits
Asano, 2016 [49]	M, 29	No	Bilateral LE pain, paraplegia, T10 sensory level, absence of anal sphincter tone, visual field deficits followed by blindness, bilateral pleural effusion	Intramedullary T6 lesion, T6-9 paravertebral mass, pituitary mass, bilateral orbital cavity masses	Yes	Yes	Not mentioned	Surgery, systemic CTX (daunorubicin plus cytarabine)	1 year	Alive. In remission, but neurologic deficits (paraplegia and blindness) persisted
Dhakar, 2016 [50]	M, 55	Yes, AML	Paraparesis	Multiple osseous and extra-osseous paravertebral masses with infiltration into the epidural space	Not mentioned	Yes	Not mentioned	None, patient went into hospice	Not mentioned	Not mentioned
Horvath, 2016 [51]	M, 66	Yes, MDS	B-symptoms, paraplegia	T1-3 epidural mass	Not mentioned	Yes	LCA+, MPO+, CD15+, CD117+, CD99+	Surgery, systemic CTX with cytarabine due to progression from MDS to AML	5 weeks	Died
Kucukapan, 2016 [52]	M, 13	No	Back pain, paraparesis, and bilateral LE paresthesias	T10-12 epidural mass	Not mentioned	Yes	Not mentioned	RT and systemic CTX (HiDAC)	10 days	Alive, total remission of spine lesion
Sahu, 2016 [53]	F, 38	Yes, CML and history of scalp MS	Quadriplegia, bowel and bladder incontinence, and T4 sensory level	T4-7 epidural mass	Not mentioned	Yes	CD13+, CD33+, CD117+, t(9;22), BCR-ABL M351T mutation	CS, RT, and TKI (nilotinib)	Not mentioned	Patient died from pneumonia-related sepsis
Yang, 2016 [54]	F, 42	Yes, AML	Paraplegia, T4 sensory level, fecal incontinence	T5-12 bilateral lateral and posterior funiculi lesions in the spinal cord	Yes	No	Not mentioned	Not mentioned	Not mentioned	Died from pneumonia
Arslantas, 2018 [55]	M, 4	No	New presentation. Paraplegia	T10-12 epidural mass	Not mentioned	Yes	Not mentioned	RT, systemic CS, and CTX (AML-BMF 2012 protocol)	Not mentioned	Alive. In remission
Koh, 2019 [56]	M, 68	Yes, CML	Back and bilateral LE pain, paraparesis, T12 sensory level, urinary retention, and anal sphincter dysfunction	T11-12 epidural and paraspinal masses	Not mentioned	Not mentioned	Not mentioned	Surgery, regional RT, and CS	35 days	Alive. Transferred to local hospital for conservative therapy
Gupta, 2020 [57]	M, 59	No	Back pain, paraplegia, bilateral LE hypoesthesia, and urinary incontinence	T7-10 and L2 epidural masses	Not mentioned	Yes	CD45+, CD117+, CD43+, MPO+, CD34-, NMP1 mutation	Surgery, systemic CTX (FLAG-IDA), and IT CTX (cytarabine)	Not mentioned	Alive. In remission
Kumar, 2020 [58]	F, 64	Yes, AML in the setting of PMF	Paraparesis, bowel and urinary incontinence	T3-10 posterior epidural masses	Not mentioned	Not mentioned	Not mentioned	Regional RT and systemic CTX	Not mentioned	Died
Bai, 2021 [59]	M, 29	No	Bilateral LE pain, dysuria	L2-L4 lumbar canal mass	Not mentioned	Not mentioned	CD33+, MPO+, partial CD68+	Surgery, systemic CTX (daunorubicin + cytarabine)	10 months	Alive. In remission
Bhandohal, 2021 [60]	M, 47	No	Back pain, left LE pain, paresis and numbness	L4-L5 intrathecal mass with invasion of L5 nerve root	Not mentioned	No	CD33+, CD34+, CD45+, MPO+, CD117+, FLI1+, CD99+	Surgery, regional RT	6 months	Alive, improvement in neurologic deficits, and decreased size of mass
Shah, 2021 [61]	M, 47	No	Back and right LE pain, anorexia, weight loss, mild sacral bulging	L4-S5 paraspinal mass	Not mentioned	No	MPO+, CD30+, CD13+	Systemic CTX (induction with cytarabine and daunorubicin, followed by consolidation with HiDAC), regional RT	Not mentioned	Not mentioned
	M, 24	No	Paraplegia with bilateral LE sensory loss	T4-7 paravertebral mass	Not mentioned	Yes (CML in chronic phase)	t(9;22), BCR-ABL rearrangement	Surgery, RT, and TKI (imatinib)	Not mentioned	Alive
	F, 32	No	Paraparesis	T6-8 paravertebral mass	Not mentioned	No	Not mentioned	Systemic CTX (daunorubicin + cytarabine), followed by surgery, local RT, and consolidation CTX (HiDAC)	2 years	Alive. In remission
Han, 2022 [62]	M, 37	Yes, CML	Back and chest pain	T2-L2 epidural mass	Not mentioned	No	CD15, CD3, CD20, MPO, CD79a	Surgery, readjustment of TKI therapy (switch from imatinib to dasatinib)	1 year	Alive. In remission
Panda, 2022 [63]	M, 13	No	Urinary retention and overflow incontinence, constipation, saddle anesthesia, loss of anal reflex	L5-S4 spinal canal mass	Not mentioned	Yes	CD34+, MPO+	Surgery, systemic CTX	Not mentioned	Alive. Receiving systemic CTX
Shao, 2022 [64]	M, 15	No	Paraparesis, T6 sensory level, back and chest pain	T3-8 spinal space-occupying mass	Not mentioned	Yes	CD13+, CD33+, CD34+, CD38+, CD56+, CD117+, HLA-DR+, MPO+	Systemic CTX (idarubicin + cytarabine)	1 year	Alive. In remission
Fujikawa, 2023 [65]	M, 14	No	Back pain, paraplegia, T9 sensory level, bladder and bowel dysfunction	T7-9 posterior epidural mass	No	Yes, AML (82.5% of myeloblasts)	MPO+, CD34+, t(8;21), RUNX1-RUNX1T1 rearrangement	Surgery, systemic CTX (cytarabine, mitoxantrone, etoposide)	8 months	Alive. In remission, incomplete paraplegia and bladder and bowel dysfunction persisted
Nair, 2023 [66]	M, 36	Yes, CML	Back pain, unsteady gait, paraparesis	T5-11 epidural, pelvic, and bilateral proximal femoral masses	Not mentioned	Not mentioned	MPO+, CD34+, CD117+	Surgery, systemic CTX	Not mentioned	Not mentioned
Osman, 2023 [67]	M, 20	Yes, AML	Back pain, paraparesis, T4 sensory level, and urinary retention	T2-6 epidural mass extending to the subdural space	Not mentioned	Not mentioned	MPO+, CD43+	CS, surgery	Not mentioned	Not mentioned
Patel, 2023 [68]	F, 87	No	Paraparesis	T4-7 epidural mass	Not mentioned	Yes	CD68+ (MS with monocytic differentiation)	Surgery, declined further CTX or RT	3 months	Died

**Table 3:** Demographics and patients' characteristics. AML: acute myeloid leukemia; BM: bone marrow; BMT: bone marrow transplant; CNS: central nervous system; CML: chronic myeloid leukemia; CTX: chemotherapy; IQR: interquartile ration; IT: intrathecal; MDS: myelodysplastic syndrome; MPN: myeloproliferative neoplasm; MS: myeloid sarcoma; PMF: primary myelofibrosis; RT: radiotherapy

Variable		
Patient number, n	69	
Gender		
Male, n (%)	55 (80)	
Age		
Age, median (IQR)	29 years (14-47)	
History of AML, MPN, or MDS		
No, n (%)	47 (68)	
Yes, n (%)	22 (32)	
AML, n (%)	14 (20)	
CML, n (%)	5 (7)	
MDS, n (%)	2 (3)	
PMF, n (%)	1 (1)	
Signs and symptoms at presentation		
Paresis/paralysis, n (%)	56 (81)	
Sensory deficits, n (%)	41 (59)	
Back pain, n (%)	41 (59)	
Bladder dysfunction, n (%)	28 (40)	
Bowel dysfunction, n (%)	18 (26)	
CNS disease at presentation		
Yes, n (%)	8 (11)	
No, n (%)	15 (22)	
Not mentioned, n (%)	46 (67)	
BM disease at presentation		
Yes, n (%)		39 (57)
No, n (%)		21 (30)
Not mentioned, n (%)		9 (13)
Treatment		
Systemic CTX		49 (71)
RT		39 (56)
Surgery		39 (56)
IT CTX		13 (19)
BMT		9 (13)
Outcomes at time of publication		
Alive, n (%)		32 (46)
Interval follow-up, median (IQR)		6 months (3-12)

Discussion

MS is a rare extramedullary manifestation of AML or other MPN and MDS that can present in virtually any anatomical location, including the spinal cord. Spinal MS, although rare, represents a critical clinical challenge due to the potential for spinal cord compression, which can lead to severe neurological deficits that might become permanent if not promptly managed. Our case report and literature review showcase these severe neurologic deficits that can arise in patients with spinal MS, such as muscle weakness, sensory deficits, and bladder and bowel dysfunction. The delay in diagnosis due to the insidious onset of symptoms emphasizes the need for high clinical suspicion, especially in patients with a history of hematologic malignancies.

MRI plays a crucial role in the diagnosis of MS, particularly in cases with spinal cord involvement. The whole spine MRI findings (intradural enhancing masses with associated nerve root thickening) were critical in establishing the diagnosis in our patient, especially in the setting of the previous history of AML. Other reported cases also highlight the importance of prompt imaging studies, MRI in particular, in patients presenting with signs of spinal cord compression, as it supports an early diagnosis and timely interventions. These imaging findings help plan therapeutic strategies, including potential surgical decompression, RT, and systemic CTX [69,70]. Despite the high yield of imaging in the diagnostic workup of MS, the definitive diagnosis often relies on a pathology assessment of a biopsy specimen.

Given its rarity and lack of RCTs, there is no consensus regarding the standard management of spinal MS; however, it generally requires a multimodal approach. According to our literature review, most patients were treated with some combination of systemic or IT CTX, RT, CS, and/or surgery. In this case, our patient received systemic and IT CTX, as well as regional spinal RT. RT and steroids may provide relief of the spinal cord compression; however, as virtually all cases with MS ultimately progress to AML, treatment protocols for AML (e.g., systemic CTX with subsequent allogeneic SCT) are usually considered the most reasonable approach [1,71]. Our patient would ultimately benefit from more CTX with a CNS-penetrating agent such as HiDAC, followed by allogeneic SCT.

The literature review highlights poor outcomes for patients with MS presenting with spinal cord compression, with only 46% of patients alive at the time of publication and none of them in remission. The median follow-up of six months reflects the aggressive nature of this disease. The rarity of MS with spinal cord compression limits the feasibility of RCTs, and current treatment recommendations rely heavily on case reports and expert opinion. This underscores the need for collaborative studies and registries to establish optimal management strategies formally. Additionally, advancements in molecular diagnostics and targeted therapies may provide opportunities for improved outcomes. Integrating novel targeted agents, such as FLT3 inhibitors or monoclonal antibodies, into treatment protocols could potentially enhance responses while mitigating toxicities.

## Conclusions

Although rare, spinal MS should be suspected in a patient with a history of myeloid neoplasia presenting with new-onset neurologic deficits, particularly if imaging shows mass lesions involving the spinal cord. Management typically involves a multimodal approach, including a combination of CTX, RT, CS, and/or surgery. Despite treatment, outcomes are poor, and further collaborative studies are essential to develop standardized management strategies and improve prognosis in this aggressive disease.
